# RNA-Seq Profiling between Commercial and Indigenous Iranian Chickens Highlights Differences in Innate Immune Gene Expression

**DOI:** 10.3390/genes14040793

**Published:** 2023-03-25

**Authors:** Ayeh Sadat Sadr, Mohammadreza Nassiri, Mostafa Ghaderi-Zefrehei, Maryam Heidari, Jacqueline Smith, Mustafa Muhaghegh Dolatabady

**Affiliations:** 1 South of Iran Aquaculture Research Institute, Iranian Fisheries Science Research Institute (IFSRI), Agricultural Research Education and Extension Organization (AREEO), Ahvaz 71867-37533, Iran; 2 Recombinant Proteins Research Group, The Research Institute of Biotechnology, Ferdowsi University of Mashhad, Mashhad 91779-48974, Iran; 3 Research Associate/Peptide Drug and Bioinformatics, School of Biotechnology and Biomolecular Sciences Level 2, E26, University of New South Wales (UNSW), Sydney, NSW 2052, Australia; 4 Department of Animal Science, Agricultural Faculty, Yasouj University, Yasouj 75918-74934, Iran; 5 Department of Animal Sciences, College of Agriculture, Isfahan University of Technology, Isfahan 83111-84156, Iran; 6 The Roslin Institute and Royal (Dick) School of Veterinary Studies, The University of Edinburgh, Easter Bush, Midlothian EH25 9RG, UK

**Keywords:** chickens, commercial, indigenous, gene expression, immune genes, RNA-Seq, transcriptome

## Abstract

The purpose of the current study was to examine transcriptomic-based profiling of differentially expressed innate immune genes between indigenous and commercial chickens. In order to compare the transcriptome profiles of the different chicken breeds, we extracted RNA from blood samples of the Isfahan indigenous chicken (as indigenous) and Ross broiler chicken (as commercial) breeds. RNA-Seq yielded totals of 36,763,939 and 31,545,002 reads for the indigenous and commercial breeds, respectively, with clean reads then aligned to the chicken reference genome (Galgal5). Overall, 1327 genes were significantly differentially expressed, of which 1013 genes were upregulated in the commercial versus the indigenous breed, while 314 were more highly expressed in the indigenous birds. Furthermore, our results demonstrated that the *SPARC, ATP6V0D2, IL4I1, SMPDL3A, ADAM7, TMCC3, ULK2, MYO6, THG1L* and *IRG1* genes were the most significantly expressed genes in the commercial birds and the *PAPPA, DUSP1, PSMD12, LHX8, IL8, TRPM2, GDAP1L1, FAM161A, ABCC2* and *ASAH2* genes were the most significant in the indigenous chickens. Of notable finding in this study was that the high-level gene expressions of heat-shock proteins (HSPs) in the indigenous breeds could serve as a guideline for future genetic improvement. This study identified genes with breed-specific expression, and comparative transcriptome analysis helped understanding of the differences in underlying genetic mechanisms between commercial and local breeds. Therefore, the current results can be used to identify candidate genes for further breed improvement.

## 1. Introduction

Chickens are a classic avian experimental model species, and they have been used extensively in developmental research [1]. The poultry industry is one of the most important players within the livestock production industries [2]. However, the poultry industry is now confronted with growing demands for food security in an increasing global population. The most important issues are control of infectious disease and food safety. Disease prevention and control are thus critical factors in this industry. The immune system plays a key role in maintaining host health and in modulating the pathogeneses of a wide range of diseases. The avian immune system includes both an innate and an adaptive arm. Innate immunity is the first line of defense against multiple types of pathogens [3]. Avian immune-related genes play an important role in resistance and susceptibility against disease in different chicken strains [4]. Selection pressure stemming from modern high-efficiency growth systems for commercial poultry has negatively affected immune competence; thereby, intensive chicken breeding has created chicken lines that are more susceptible to common diseases [5,6]. An effective selection program for the poultry industry would be based more on effective innate immune responses as compared to acquired responses. Selection of poultry based on effective innate immune responses will produce a population of birds with multivalent protection against diverse pathogens and increased survival in the field [7]. Indigenous breeds of chickens play an important role in rural economies in most developing and underdeveloped countries. Indigenous chicken populations are an invaluable genetic resource as a consequence of initial selection practices based on specific morphological features. However, they do tend to show low productivity and need to be bred for improvement [8]. They are also excellent animal models for studying natural immunity against common diseases. Increased genetic resistance to disease and adaptation to stressful environments in many indigenous breeds are notable and remarkable traits. Genetic selection can improve disease resistance, with resistance able to develop through indirect selection, primarily in immune response traits [9,10]. Host immune responses are genetically variable from breed to breed and are related to the complex mechanisms and specific organs that define the strategies and types of cells that have roles against multiple types of pathogens. Therefore, efforts to select poultry with efficient innate immune responses would be beneficial for genetic selection strategies. Gene expression, or transcriptome analysis, is a major area of functional genomics. This technique provides a valuable approach for a better understanding of the specificities of cells, tissues and organs at the transcriptional level. Next-generation sequencing (NGS) enables identification of expressed transcripts with or without prior knowledge of gene sequences and has become a powerful tool to investigate the transcriptional profiles of many organisms [11]. RNA sequencing (RNA-Seq) is the technology used to identify differentially expressed transcripts and pathways correlated with different conditions and to explore unknown genes [12]. Therefore, this analytical method can be applied to understanding the molecular basis of the chicken immune system. Several studies have been performed to investigate gene expression profiles for disease resistance in chickens. For instance, in order to analyze disease resistance, Dar et al. compared commercial broilers and a local chicken line (Kashmir faverolla) and reported that interleukins, cytokines, *NOS2*, Av-defensins, toll-like receptors and other immune-related gene families were among the genes that showed differential expression. The Kashmir faverolla line was considerably enriched regarding the majority of the genes and signaling pathways involved in the innate and adaptive immune responses against bacterial infection [13]. There is still a lack of knowledge on host innate immune reactions to infection with pathogens of variable virulence, as well as how these reactions may contribute to serious pathological harm and mortality upon infection with highly virulent strains. Cheng et al. used RNA-Seq transcriptional profiling of chicken embryonic visceral tissues (CEVTs) infected with either the highly virulent NA-1 Newcastle disease virus strain or the avirulent LaSota vaccine strain to examine the differences in epigenetic and pathogenesis mechanisms between the different strains [14]. Despite these studies, further research on the molecular mechanisms of resistance to diseases in poultry is needed. Specifically, there is a lack of information related to the gene-expression-profile differences between indigenous and commercial chicken breeds. RNA-Seq can help us to understand this question [15]. The study of different breeds of poultry from different geographical locations has been reported [16]. In this study, we implemented an RNA-Seq approach to compare transcriptome profiling of two chicken breeds. Ross birds represent the leading global broiler brand and were used here as a representation of highly selected commercial birds. Isfahan birds (with high residual feed intake) were chosen as an example of a local indigenous breed of chicken that has not been subject to selection and lives under wild, scavenging conditions. These two populations represent two extremes, allowing us to examine the fundamental differences in the immune system caused by the differences in the two lifestyle histories. Our goal was to better understand the biological and molecular mechanisms involved in the immune system between indigenous and commercial chicken breeds and establish a foundation for developing improved breeding strategies.

## 2. Materials and Methods

### 2.1. Ethics Statement

All animal experiments were performed in accordance with the relevant guidelines and regulations, with approvals from Iran’s Environmental Protection Authority and the Ferdowsi University of Mashhad Animal Ethics Committee, in line with the 1964 Helsinki declaration and its later amendments or comparable ethical standards. This study was carried out in compliance with the ARRIVE guidelines.

### 2.2. Animals, RNA Extraction and Sequencing

The chickens used for this study were from the indigenous Isfahan breed and the Ross breed and were raised on the farm of the Safi-Abad Agriculture and Education Centre, Dezful, Iran. These birds were kept under the same environmental and nutritional conditions. Six chickens (47 days of age) were selected randomly from each breed, and blood samples were collected from the brachial/ulnar wing vein. The total RNA was extracted using Trizol (Invitrogen, Waltham, MA, USA) according to the manufacturer’s protocol. The quality and quantity of the RNA were assessed with NanoDrop (Thermo Scientific™ NanoDrop 2000, Waltham, MA, USA) and gel electrophoresis. The 28S/18S ratio for the RNA samples ranged from 1.8 to 2. The RNA integrity numbers (RINs) for the total RNA were >8.0 for each sample. The total RNA was sent to BGI Genomics (Shenzhen, China) for sequencing. For each breed, three RNA samples were pooled (via mixing equal quantities of RNA) and split into 2 libraries (representing 4 libraries in total). The four RNA-Seq libraries were sequenced based on Illumina HiSeq 2000 protocols to generate 150 bp paired-end reads.

### 2.3. Data Quality Control, Read Mapping and Transcriptome Analysis

Preliminary quality control analysis of the raw data was checked with FastQC (v0.11.5) [17]. Raw reads were processed for library adapter removal and low-quality reads filtered using Trimmomatic (v0.35) with the following parameters: ILLUMINACLIP: adapter. fasta: 2:30:10; HEADCROP:5; LEADING:25; TRAILING:25; MAXINFO: 50:0.95 and MINLEN:50 [18]. The reference chicken genome (Galgal5) and the annotation GTF file were downloaded from the Ensembl repository (http://asia.ensembl.org/info/data/ftp/index.html (accessed on 11 June 2018)). The cleaned reads were mapped to the reference genome using HISAT2 (v2-2.0.3) (http://ccb.jhu.edu/software/hisat2/index.shtml (accessed on 3 July 2018)) [19]. Cufflinks (v2.2.1) was used to assemble the aligned reads for each sample into transcripts with HISAT2 and estimate the gene expression of each transcript via assigning an FPKM (fragments per kilo base of exon per million reads) value [20]. Here, abundances of transcripts were upper-quartile normalized. Then, all the assembled transcripts generated with Cufflinks were merged to generate unique transcripts using the Cuffmerge tool. This approach maximized the overall quality of the final assembly. Differential expression of annotated genes was performed using Cuffdiff. To improve the differential expression tests for less-abundant transcripts, the upper-quartile normalization option was chosen. Finally, any gene with a false discovery rate (FDR) of ≤ 0.05 and a log2FC value where log2FC > 0.8 and log2FC < −0.8 was considered a differentially expressed gene (DEG) between the two breeds.

### 2.4. Functional Annotation

To test for gene set enrichment analysis, gene ontology (GO) terms and Kyoto Encyclopedia of Genes and Genomes (KEGG) pathways were identified using the Database for Annotation, Visualization and Integrated Discovery (DAVID) tool (https://david.ncifcrf.gov/ (accessed on 19 November 2018)) [21]. Briefly, we investigated which functional terms or pathways were annotated to at least two genes and were statistically significant in the study gene set. The calculated *p*-values were corrected through the Benjamini–Hochberg procedure, taking corrected *p*-values of ≤ 0.05 as a threshold of significance. Ingenuity pathway analysis (IPA) software (Qiagen—https://digitalinsights.qiagen.com/products-overview/discovery-insights-portfolio/analysis-and-visualization/qiagen-ipa/ (accessed on 19 December 2022)) was used to reveal biological pathways and functions pertaining to the DEGs identified in our analyses. The *p*-values were calculated using the right-tailed Fisher Exact Test (threshold *p* < 0.05). Enriched miRNA targets and transcription-factor binding sites were identified using WebGestalt (http://www.webgestalt.org/ (accessed on 19 December 2022)) [22]. An over-representation analysis (ORA) was carried out using the network functional database to identify both transcription-factor and miRNA targets. The ‘chicken genome’ was used as the background reference. Results showing FDRs of < 0.05 were considered significant.

### 2.5. Protein–Protein Interaction (PPI) Network

To determine the functional relationship among immune-related genes, DEGs (with corrected *p-*values of ≤ 0.05), which were annotated for the immune-system process, were imported to the search tool for retrieval of interacting genes/proteins (STRING v10.0, http://string-db.org/ (accessed on 7 March 2019)) via selecting *Gallus gallus* as a model organism. STRING could identify a network of close interactions among this set of genes based on databases of experimental as well as predicted protein interactions [23]. We discarded PPIs with confidence scores of <0.4 (a commonly used threshold). Disconnected nodes were hidden. Three calculation methods of network topological features were used to analyze the importance of the nodes in the PPI network. These three methods were degree centrality, betweenness centrality and closeness centrality, which were used to calculate the topology scores of the nodes in the PPI network using the CytoNCA plugin in Cytoscape [24]. Nodes with higher scores may play important roles in a PPI network and are considered hub nodes. The MCODE plugin in Cytoscape was used to analyze the subnetwork module in the PPI network, with a module score of >4.5. In MCODE, each module has a score. The higher the score, the closer the interaction among the nodes in the module [25].

### 2.6. Real-Time PCR

To confirm the DEGs with real-time RT-PCR, the same RNA samples used for sequencing from the two breeds (6 samples per breed) were used. The RNA concentrations were measured using a NanoDrop 2000 Spectrophotometer (Thermo Scientific™). Reverse transcription was conducted to generate cDNA libraries using a Thermo RevertAid™ First Strand cDNA Synthesis Kit, following the manufacturer’s instructions. Real-time PCR was performed using a LightCycler^®^ 96 instrument (Roche, Germany). The sequences of all primers used are listed in Table 1 and were designed with the PRIMER3 program (http://frodo.wi.mit.edu (accessed on 16 September 2020)). qPCR reactions were performed in triplicate, and the average cycle threshold (Ct) values were determined for each sample. The real-time qPCR amplification conditions were as follows: one cycle at 95 °C for 2 min and 40 cycles each at 95 °C for 30 s and 60 °C for 30 s. The chicken β actin (*ACTB*) gene was used as an internal control. The results were analyzed using the 2^−ΔΔCt^ method [26].

## 3. Results

### 3.1. Quality Control and Mapping of RNA-Seq Data

**Figure 1:**
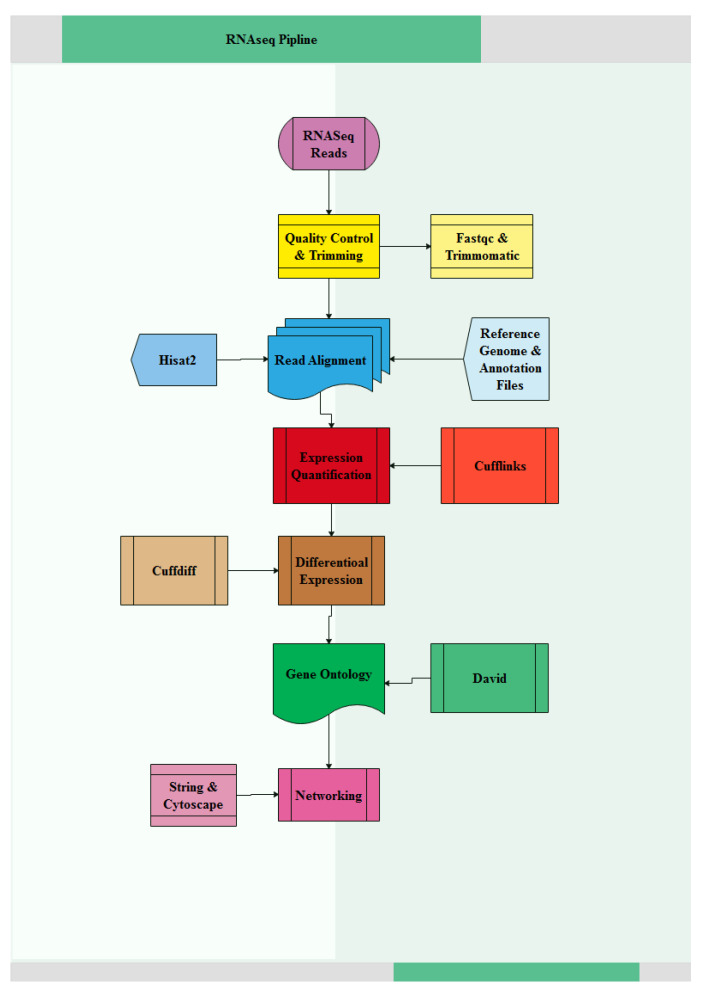
The methodology pipeline used in this study.

The experimental pipeline used in this study is shown in Figure 1. RNA-Seq was performed on the Illumina HiSeq 2000 platform and yielded totals of 36,763,939 and 31,545,002 reads for the indigenous and commercial breeds, respectively. Following adaptor removal, quality filtering and removal of too-short sequences, only a few reads (<0.02%) did not pass quality filtering. The cleaned reads were then aligned to the chicken reference genome (Galgal5). Approximately 85% of sequenced fragments were aligned successfully to the reference genome, with no more than two mismatches allowed. The numbers of total raw and trimmed fragments and alignment rates for each sample are summarized in Table 2.

### 3.2. Gene Expression Analysis

**Figure 2:**
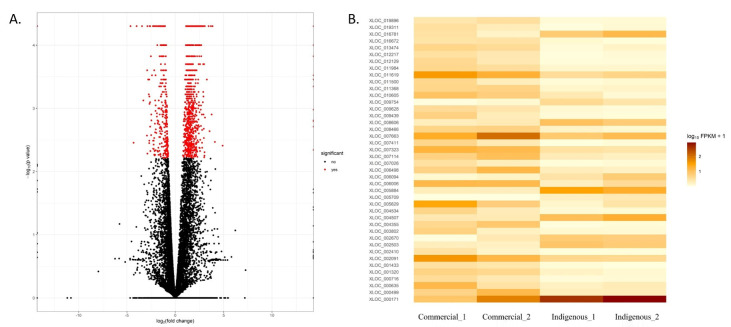
Analysis of differentially expressed genes (DEGs) between commercial and indigenous birds. (**A**) Log2-fold change between the commercial versus indigenous (native) breed. Light blue dots represent significant DEGs between breeds (*p*-values < 0.05). (**B**) Heat map of differentially expressed genes between the commercial versus indigenous breed. Lighter color represents lower expression.

Gene expression levels were estimated using the FPKM method, and 20,034 expressed genes were detected. Of these, 3381 were novel and 16,653 were known genes. Among the evaluated genes for gene expression analysis, 1327 genes were significantly differentially expressed between the two breeds (false discovery rate (FDR) < 0.05) (Figure 2A). Of the 1327 differentially expressed genes (DEGs), 1013 showed higher expressions in the commercial birds compared to 314 genes with higher expressions in the indigenous breed (Figure 2B). In addition, 153 unannotated genes were identified as differentially expressed: 73 up- and 56 downregulated in the commercial breed compared with the indigenous breed. The top 10 annotated up- and downregulated genes in the local breed are presented in Table 3. Moreover, the details of all DEGs are represented in Supplementary Table S1. Many DEGs are directly involved in the immune system; all these genes were upregulated in the commercial versus the indigenous breed.

### 3.3. Confirmation of Differential Gene Expression with Real-Time PCR

**Figure 3:**
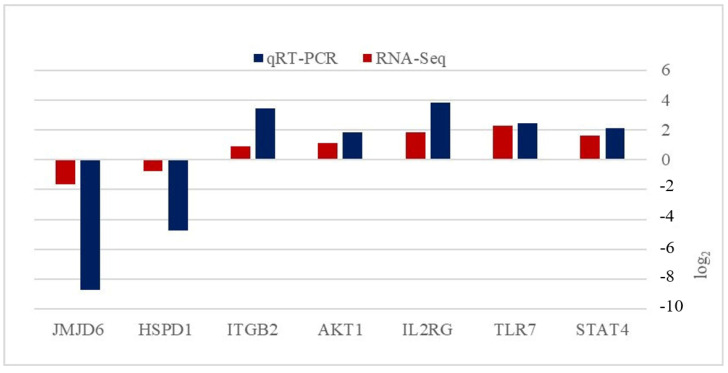
Fold-expression changes measured with RNA-Seq and real-time RT-PCR.

To technically validate the RNA-Seq results, seven differentially expressed genes (five upregulated genes from the innate immune system and two random downregulated genes) were selected for real-time PCR analysis. The correlation between the mRNA expression levels from real-time PCR and RNA-Seq was relatively high, and the expressions of genes were correlated with the transcriptome data, indicating the reliability of the data (Figure 3).

### 3.4. Functional Annotation of Differentially Expressed Genes

**Figure 4:**
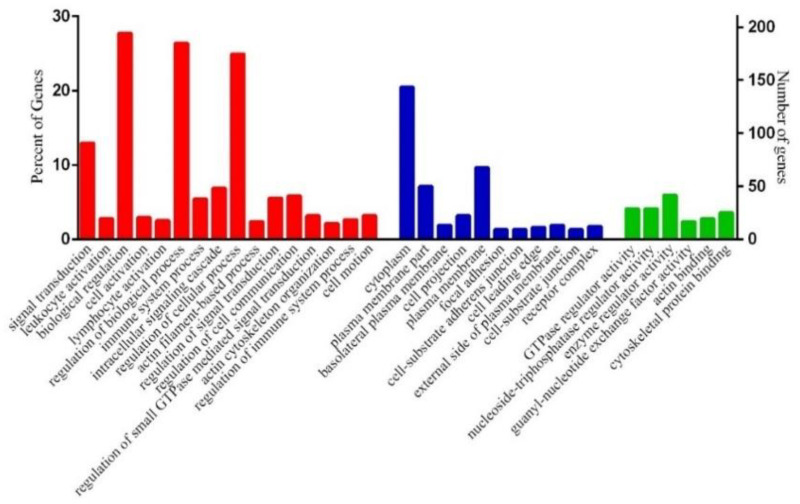
Histogram of gene ontology (GO) classification. The terms are summarized in three categories, biological process (red), cellular component (blue) and molecular function (green), for upregulated DEGs (corrected *p-*values ≤ 0.05).

**Figure 5:**
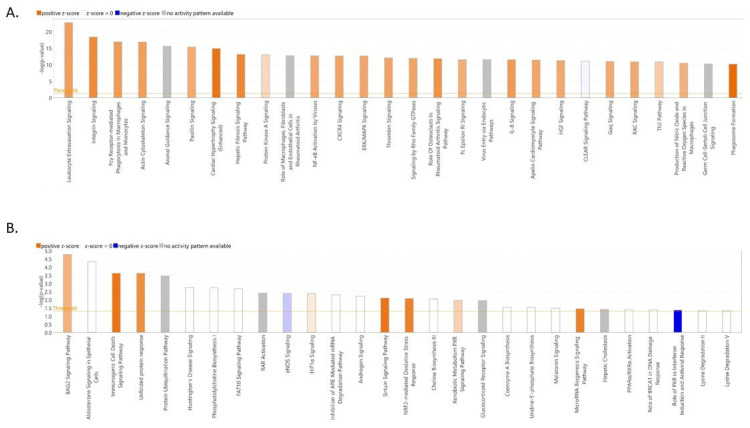
Ingenuity pathway analysis. (**A**) The biological pathways highlighted in gene expression in commercial birds. (**B**) The biological pathways highlighted in gene expression in indigenous birds. Orange—pathway activation; blue—pathway repression.

To gain insight into the functionality of the DEGs in the blood samples associated with the two chicken breeds, we performed a gene set enrichment analysis. The DAVID functional enrichment analysis revealed that there were 54 significant biological terms for the 1013 DEGs that were more highly expressed in the commercial birds. Interestingly, most of these biological-process terms were related to the immune system, including leukocyte activation, lymphocyte activation, immune-system process, regulation of immune-system process, leukocyte differentiation and immune-system development (Figure 4). Pathway analysis was also carried out to investigate potential gene function. Eighteen different Kyoto Encyclopedia of Genes and Genomes (KEGG) pathways showed significant enrichment when upregulated DEGs were linked to gene ontology (GO) and KEGG annotations. We found that the most significant pathways and functional categories were involved in the immune system, particularly the innate immune system (Table 4). These enriched functional terms and pathways could give us more insights into potential differences in molecular mechanisms of resistance to diseases between commercial and indigenous birds. On the other hand, genes more highly expressed in the indigenous birds were significantly enriched for just one biological-process term (protein folding). In addition, KEGG pathway analysis results showed that no pathway was found considerably enriched for the genes in this gene set. The details of the significant GO terms and KEGG pathways in the two comparison groups are presented in Supplementary Table S2. When specific pathways were analyzed in detail using ingenuity pathway analysis, we could see that the immune functions highlighted in gene expression in the commercial birds included leukocyte activation, phagocytosis, integrin signaling, NF-kB activation and IL8 signaling (Figure 5A). Interestingly, in the indigenous birds, we did not see this immune gene expression signature. However, the most significantly enriched pathway was that of BAG2 signaling. Also highlighted were the sirtuin signaling pathway and the NRF2-mediated oxidative stress response (Figure 5B). We were also able to examine whether the identified DEGs were enriched as targets for particular regulatory factors such as transcription factors or miRNA. Through carrying out an over-representation analysis within the WebGestalt package, we could see that this was indeed the case for the genes that were more highly expressed in the commercial birds. Supplementary Table S3 shows that DEGs were enriched in binding sites for transcription factors including ETS, PU1, ELF1, STAT6 and IRF1. Meanwhile, as shown in Supplementary Table S4, many of these DEGs were also targets for gga-mir124A, gga-mir506 and gga-mir155.

### 3.5. PPI Network of Immune-System-Related Genes

**Figure 6:**
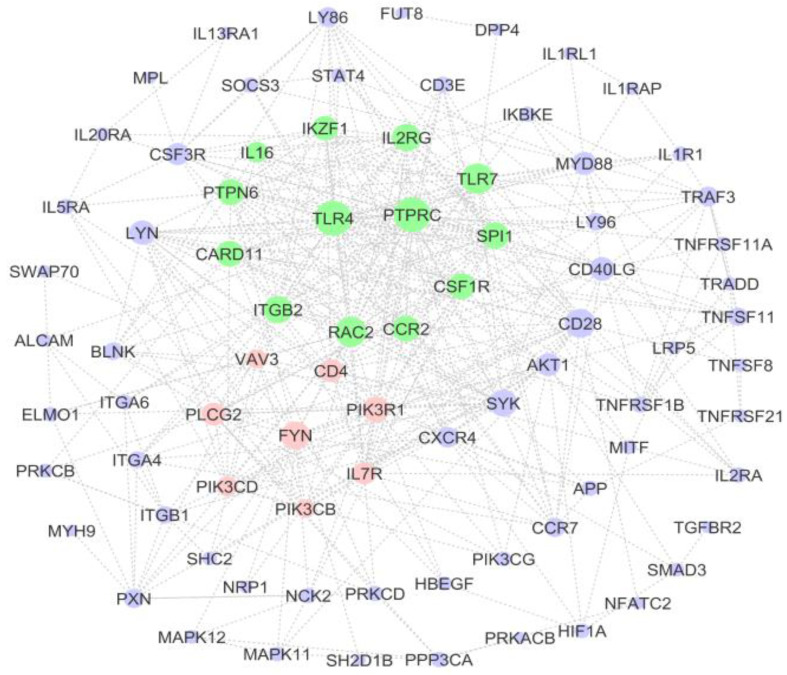
PPI network of immune-system-related upregulated DEGs. The size of each node is based on degree value; a bigger size is related to a larger degree value. The color of each node is related to one of two subnetwork modules that were obtained (green and pink).

STRING analysis indicated that of the 87 input proteins, 69 were identified in the database and were used to construct the PPI network (Figure 6). The statistical enrichment analysis incorporated in STRING revealed that the PPI network was significantly enriched. The nodes with the top 10 highest topology scores, which were calculated with three centrality methods, are shown in Table 5 and can be considered hub nodes. Toll-like receptor 4 (TLR4) and protein tyrosine phosphatase receptor type C (PTPRC) had the highest scores in the three centrality methods (Table 5). Based on the threshold of a module score of >4.5, two subnetwork modules were obtained. The green cluster consisted of thirteen nodes (genes) and 78 edges (connections) with an MCODE score of 13, while the pink cluster consisted of eight nodes and 18 edges with an MCODE score of 5.14 (Figure 6). The module was significantly enriched for 26 GO terms, including regulation of immune-system process and regulation of lymphocyte activation pathway.

## 4. Discussion

The immune systems of livestock directly affect health and are influenced by genetics. Health status and immune robustness play important roles in farm profitability [27]. RNA-Seq allows detection of the key genes associated with important traits such as these [28]. Therefore, high throughput sequencing was performed to evaluate the gene expression profiles of two chicken breeds with extreme phenotypes to understand potential immune-system differences that could play key roles in health maintenance and pathogenesis of disease. Our findings showed that innate immune-related genes were upregulated in the commercial birds, with gene ontology analysis verifying GO terms related to the innate immune system in this group. The regulatory mechanisms working on the genes that were more highly expressed in the commercial birds are also known to be involved in immune-system processes. For example, many DEGs were seen to harbor binding sites for particular transcription factors. These included STAT6, which is involved in IL4 responses, [29] and IRF1, which has a key role in the antiviral response [30]. Similarly, many of these genes are targets for gga-mir-155, a microRNA variety already known to be implicated in various immune responses [31,32]. Based on previous studies, selection for accelerated growth strongly and significantly reduces the host responses to a variety of immune challenges [33,34,35]. Selection pressure plays a key role in accelerated growth, but on the other hand, it significantly compromises the host immune system.

Upon examination of the downregulated DEGs, no strong expression of immune genes was seen. However, heat-shock proteins were seen to be more highly expressed in the indigenous chickens. Heat-shock proteins are induced in response to environmental, physical and chemical stress, limiting the consequences of damage and facilitating cellular recovery. One of the most important results of this study is the high expression level of heat-shock protein (HSP) genes in the indigenous birds compared with that of the commercial breed. Based on the encoded proteins’ approximate relative molecular masses, HSPs are categorized as HSP100, HSP90, HSP70, HSP60 and small HSP. The HSP70 family is the most conserved family and has been the most intensely studied in various organisms [36,37]. Of the 11 HSP genes showing differential expressions between the two breeds in this study, half of them belonged to HSP70. Due to the high tolerance of indigenous breeds to challenging environmental conditions and poor breeding methods, it seems that higher HSP expression in the indigenous breed increases their resistance to environmental stress. These proteins are considered a first line of defense against environmental stress. Hence, based on these results, we infer that overexpression of these genes could lead to enhanced survivability and stress tolerance of the cells in the indigenous breeds. Pathway analysis also showed, in particular, BAG2 and sirtuin signaling to be important, along with the NRF2-mediated response to oxidative stress. BAG2 binds to the HSC70/HSP70 complex and is involved in the cellular response to heat stress [38]. Sirtuins are involved in metabolic regulation and also help regulate heat stress [39]. Oxidative stress can occur through means of nutritional stress, heat stress and pathogenic stress, so it is interesting to note the activation of the NRF2-mediated pathway in the local birds, which is very likely a means of overcoming the harsh environmental conditions these birds face.

Pathway analysis indicated the importance of the Toll-like receptor (TLR) pathway in our comparison. TLRs are transmembrane proteins involved in recognition of pathogen-associated molecular patterns (PAMPs) such as lipopolysaccharides, lipopeptides, peptidoglycans, flagellin, bacterial DNA and viral double-stranded RNA. The majority of similar studies have described the role of the TLR family, which plays an important role in innate immunity [40]. The genes related to this specific family are responsible for detecting microbial pathogens and generating innate immune responses. The *PIK3CG, PIK3CB, LY96, PIK3CD, MAPK11, TLR4, TLR7, TLR2-1, AKT1, IKBKE, STAT4, MYD88, MAPK12, PIK3R1* and *TRAF3* genes, which belong to this family, were more highly expressed in the commercial breed. Myd88 is a central node of the Toll-like receptor signaling pathway and can interact with various TLRs, such as TLR-2, TLR4 and TLR7. Higher TLR mRNA levels were seen in the commercial birds compared to in the indigenous birds. Amongst the TLRs, *TLR7* showed higher expression than the others. Chicken TLR7 has been reported to respond to R848, Poly (I:C), loxoribine and imidazoquinoline compounds, which have antiviral activity. Furthermore, its role in intracellular recognition of nucleic acids has been demonstrated; thus, higher *TLR7* expression might provide improved protection against viral diseases [40]. Previous studies have already shown differential expression of TLRs between commercial and indigenous chickens [41]. Another important component of immunity is the cytokines (soluble extracellular proteins and glycoproteins) that are crucial intercellular regulators and mobilizers of cells engaged in innate as well as adaptive inflammatory host defenses, cell growth, differentiation, cell death, angiogenesis and development and repair processes aimed at restoration of homeostasis [42]. Due to the importance of chicken cytokines in disease prevention, we were interested in looking at this group of genes specifically. In this study, we identified some of the genes related to this family, and they were seen to be upregulated in the commercial birds compared to in the indigenous chickens. These include genes belonging to the TNF, TGF, CXC, CC and IL subfamilies (*TNFRSF21, IL1R1, IL2RA, TGFBR2, IL7R, TNFSF8, CCR7, TNFRSF1B, TNFRSF11A, TNFSF11, CCR5, CD40LG, IL20RA, CXCR4, CCR2, IL1RAP, CSF3R, CSF2RB, IL2RG, IL5RA, MPL* and *IL13RA1*). In addition, the *CCR2, CCR5, CCR7* and *CXCR4* genes from the chemokine signaling pathway were seen to interact with each other and comprise a gene network. Two genes related to this group were also identified—interleukins 8 and 15 (*IL8, IL15*), which were significantly upregulated in the indigenous breed.

Natural killer (NK) cells are lymphocytes of the innate immune system that are involved in early defense against both allogeneic (non-self) and autologous cells undergoing various forms of stress, such as infection with viruses, bacteria or parasites or malignant transformation [43]. In this study, natural killer (NK) cells comprised one of the top three significant pathways, with 16 genes (*PIK3CG, PTPN6, VAV3, PIK3CB, PIK3CD, ITGB2, PRKCB, RAC2, FYN, PLCG2, PPP3CA, NFATC2, SH2D1B, SHC2, PIK3R1, SYK*) that were upregulated in the commercial breed. Among the signal transduction pathways, the JAK-STAT pathway is mainly expressed in white blood cells and is involved in regulation of the immune system [44]. Our findings showed that the genes belonging to this pathway, *PIK3CG, PTPN6, IL2RA, PIK3CB, SOCS3, PIK3CD, IL7R, AKT1, STAT4, IL20RA, CSF3R, CSF2RB, IL2RG, IL5RA, MPL, IL13RA1* and *PIK3R1*, were significantly upregulated in the commercial chickens in comparison to in the indigenous birds. In the present study, SOCS3 was strongly linked with many IL receptors of the cytokine–cytokine receptor interaction pathway, indicating that all of these genes interact with each other. Apoptosis is an essential part of normal development in multicellular organisms, but it is also induced through disease [45]. In the present study, 12 genes associated with this pathway were seen to be upregulated in the commercial breed compared to in the indigenous one. According to the high-level expressions of innate immune-system genes in the commercial breed, it can be inferred that selective breeding of commercial birds has led to increased expression of immune-system genes. In knowing the local birds to be more resilient to disease than commercial birds are, this indicates that we measured activation of the immune systems in these commercial birds, which required a higher basal level of immune gene expression compared to their more robust indigenous counterparts, in which there is simply not the same level of required response.

Through analyzing the hub genes and constructing a protein–protein interaction network for differentially expressed, upregulated genes, we found that some key genes—*PTPN6* from the JAK-STAT pathway and *FYN* from the natural killer (NK) cells—were selected with higher topology scores. The protein encoded with *PTPN6* is a member of the protein tyrosine phosphatase (PTP) family. PTPs are known to be signaling molecules that regulate a variety of cellular processes [46]. FYN (FYN proto-oncogene, Src-family tyrosine kinase) is a member of the protein–tyrosine kinase oncogene family that plays roles in many biological processes, including regulating cell growth and survival, cell adhesion, integrin-mediated signaling, cytoskeletal remodeling, cell motility and the immune response [47]. As well as the strong signals from the immune-system processes, some other interesting pathways were highlighted in the commercial birds. When highly expressed genes were examined with ingenuity pathway analysis, a couple of other biological mechanisms stood out: cardiac hypertrophy signaling and hepatic fibrosis signaling. Cardiac hypertrophy is associated with rapid growth and pulmonary hypertension [48]—something that has to be closely monitored and mitigated in the broiler industry. Likewise, hepatic fibrosis has been associated with other broiler pathologies, such as woody breast syndrome [49].

Developing a novel selection program, based on an effective innate immune system, for the poultry industry has been a fundamental research principle. Selection of poultry based on a robust innate immune response would produce a population of birds with multivalent protection against diverse pathogens and increased survivability in the field. In addition, the genetic background of a breed plays an important role in regulation of the innate immune system [50]. To date, no prior comparison or differential expression analysis of immune system genes between the two different chicken breeds has been reported. The current study is one of the first comparisons of immune gene expression between commercial and local birds, and our study can help the candidate genes involved in immune-system pathways under normal conditions be understood.

## 5. Conclusions

To our knowledge, this study is the first to shed some light on the differences in host immune gene expression between commercial and indigenous chickens. The results showed genes with breed-specific expression, and the comparative transcriptome analysis helped us to understand the differences in the genetic mechanisms. The different genetic backgrounds between the two groups of chicken may have played an important role in immune competence. Likewise, characterization and evaluation of immune parameters in various genotypes can offer knowledge that can be incorporated into breeding programs to enhance natural resistance to disease. Intensive selection for improved growth and other production traits is known to come at a cost to immune fitness in commercial birds. Harnessing the genetic potential of local breeds, which have high natural resistance to disease and adaptation to extreme environments, will enable selective breeding for birds that are both highly productive and immunologically robust. The high levels of expression of heat-shock proteins in the indigenous breeds could also serve as a guideline for future genetic improvement for environmental adaptation: a trait that is becoming increasingly important in light of the increasing global temperatures we all now face. As the global population continues to expand and the need for food security intensifies, it is becoming critical that we utilize the power of genetics to breed livestock that are more productive, healthier and more suited to their changing environments.

## Figures and Tables

**Table 1:** List of primers used for RT-PCR assay.

Gene Name	Primer Sequence	Product Size
*STAT4*	F-ttggcaaacactacagctgtc R-atggagaatgtgggtctgtag	132
*TLR7*	F-gaatgggtgatgacagaattgg R-gctgaatgctctgggaaagg	130
*IL2RG*	F-caaccccagcaagaacttcg R-tggcagatgctttcactgtag	123
*AKT1*	F-gtacctccatttaagccacaag R-acaatccatgctgtcatcttgg	117
*ITGB2*	F-acaacagctcagtcatctgc R-gttgtcacagtcgcagaagg	116
*HSPD1*	F-aagttggctatgatgcgatgc R-ttcagtcactactgcttctgc	146
*JMJD6*	F-ttccagctcctcgagttcc R-tatcaccgttacccatcatgc	125
*β–actin*	F-gaactccctgatggtcagg R-catggataccacaggactcc	106

**Table 2:** Summary of sequence-read alignments to the reference genome.

Sample Name	Raw Fragments	Trimmed Fragments	Overall Alignment Rate (%)
Indigenous Breed 1	18768307	18509420	86.68
Indigenous Breed 2	17995632	17650558	84.89
Commercial Breed 1	15923838	15677357	84.36
Commercial Breed 2	15621164	15313649	84.17

**Table 3:** Upregulated and downregulated genes between indigenous and commercial birds (upregulated genes were more highly expressed in commercial birds).

Upregulated DEG			Downregulated DEG		
Gene Name	Fold Change	FDR	Gene Name	Fold Change	FDR
*SPARC*	4.88976	0.0039	*PAPPA*	−4.31828	0.03474
*ATP6V0D2*	4.09016	0.0034	*DUSP1*	−3.44519	0.001678
*IL4I1*	3.8447	5.00 × 10^−5^	*PSMD12*	−2.91163	0.00565
*SMPDL3A*	3.75038	0.00125	*LHX8*	−2.88599	0.001678
*ADAM7*	3.67512	5.00 × 10^−5^	*IL8*	−2.70154	0.007142
*TMCC3*	3.54063	0.0023	*TRPM2*	−2.64277	0.001678
*ULK2*	3.43567	5.00 × 10^−5^	*GDAP1L1*	−2.61655	0.012906
*MYO6*	3.25879	0.00035	*FAM161A*	−2.60569	0.001678
*THG1L*	3.06427	0.0014	*ABCC2*	−2.47906	0.020111
*IRG1*	3.02656	5.00 × 10^−5^	*ASAH2*	−2.41558	0.006498

**Table 4:** Most significantly enriched KEGG pathways.

Pathway	Pathway Definition	Count	*p*-Value	List of Genes
gga04650	Natural killer cell mediated cytotoxicity	16	2.73 × 10^−4^	*PIK3CG, PTPN6, VAV3, PIK3CB, PIK3CD, ITGB2, PRKCB, RAC2, FYN, PLCG2, PPP3CA, NFATC2, SH2D1B, SHC2, PIK3R1, SYK*
gga04060	Cytokine–cytokine receptor interaction	23	0.00287	*TNFRSF21, IL1R1, IL2RA, TGFBR2, IL7R, TNFSF8, CCR7, TNFRSF1B, TNFRSF11A, TNFSF11, CCR5, CD40LG, IL20RA, CXCR4, CCR2, IL1RAP, CSF3R, CSF2RB, IL2RG, IL5RA, MPL, IL13RA1, CSF1R*
gga04620	Toll-like receptor signaling pathway	15	0.00404	*PIK3CG, PIK3CB, LY96, PIK3CD, MAPK11, TLR4, TLR7, TLR2-1, AKT1, IKBKE, STAT4, MYD88, MAPK12, PIK3R1, TRAF3*
gga04630	Jak-STAT signaling pathway	17	0.026406	*PIK3CG, PTPN6, IL2RA, PIK3CB, SOCS3, PIK3CD, IL7R, AKT1, STAT4, IL20RA, CSF3R, CSF2RB, IL2RG, IL5RA, MPL, IL13RA1, PIK3R1*
gga04210	Apoptosis	12	0.034505	*PIK3CG, AKT1, IL1R1, MYD88, PIK3CB, IL1RAP, PIK3CD, CSF2RB, PPP3CA, PRKACB, PIK3R1, TRADD*

**Table 5:** The nodes with the top 10 highest topology scores were calculated with three centrality methods.

Gene	Degree	Betweenness	Closeness
*TLR4*	32	669.87	0.59
*PTPRC*	32	665.01	0.59
*TLR7*	26	478.68	0.54
*RAC2*	25	338.41	0.54
*CD28*	24	479.34	0.55
*IL2RG*	22	295.60	0.52
*FYN*	22	612.12	0.52
*ITGB2*	22	279.22	0.52
*SYK*	21	264.84	0.53
*SPI1*	21	127.23	0.52

## Data Availability

All the Illumina sequencing reads have been deposited in the NCBI Short Read Archive (SRA) under accession number BioProject ID: PRJNA746088.

## Supplementary Materials

The following supporting information can be downloaded at: https://www.mdpi.com/article/10.3390/genes14040793/s1, Table S1: All significant DEGs upon comparison of commercial and indigenous breeds; Table S2: The significant GO terms and KEGG pathways in the two comparison groups; Table S3: Transcription factors targeting genes more highly expressed in commercial birds; Table S4: microRNAs targeting genes more highly expressed in commercial birds.
